# The Effect of Cu:Ag Atomic Ratio on the Properties of Sputtered Cu–Ag Alloy Thin Films

**DOI:** 10.3390/ma9110914

**Published:** 2016-11-10

**Authors:** Janghsing Hsieh, Shunyang Hung

**Affiliations:** 1 Department of Materials Engineering, Ming Chi University of Technology, New Taipei City 24301, Taiwan; showjessica@hotmail.com; 2 Center for Thin Film Technologies and Applications, Ming Chi University of Technology, New Taipei City 24301, Taiwan; 3 Department of Electronic Engineering, Chang Gung University, Taoyuen 33302, Taiwan

**Keywords:** Cu–Ag alloy thin films, sputtering, resistivity, structure, dissolution

## Abstract

Cu–Ag thin films with various atomic ratios were prepared using a co-sputtering technique, followed by rapid thermal annealing at various temperatures. The films’ structural, mechanical, and electrical properties were then characterized using X-ray diffractometry (XRD), atomic force microscopy (AFM), FESEM, nano-indentation, and TEM as functions of compositions and annealing conditions. In the as-deposited condition, the structure of these films transformed from a one-phase to a dual-phase state, and the resistivity shows a twin-peak pattern, which can be explained in part by Nordheim’s Rule and the miscibility gap of Cu–Ag alloy. After being annealed, the films’ resistivity followed the mixture rule in general, mainly due to the formation of a dual-phase structure containing Ag-rich and Cu-rich phases. The surface morphology and structure also varied as compositions and annealing conditions changed. The recrystallization of these films varied depending on Ag–Cu compositions. The annealed films composed of 40 at % to 60 at % Cu had higher hardness and lower roughness than those with other compositions. Particularly, the Cu_50_Ag_50_ film had the highest hardness after being annealed. From the dissolution testing, it was found that the Cu-ion concentration was about 40 times higher than that of Ag. The galvanic effect and over-saturated state could be the cause of the accelerated Cu dissolution and the reduced dissolution of the Ag.

## 1. Introduction

Cu–Ag alloys have recently been considered an alternative material for interconnections in microelectronic circuits and have been used in high-field magnets [1,2]. Their nanoparticles show excellent optical, electronic, and catalytic properties [3]. For modern ultra-large-scale-integration interconnect applications, Cu–Ag alloys can be used to increase reliability. This can be achieved by an improved interconnect architecture and by tailoring the copper microstructure in terms of low-grain boundary and interface diffusion, low electrical resistivity, and high mechanical strength [4]. A promising material modification is to fully use their alloying effects [5], while keeping electrical resistance sufficiently low. However, alloying additions may normally cause negative impacts over one or more of the critical properties that include electrical resistivity, grain size, thermal stability, texture, and surface roughness. Therefore, if Cu–Ag alloys are to be used as satisfactory interconnections in modern microelectronic devices, one must prove that one of these alloys can provide an increased resistance against electromigration, improved mechanical properties, improved reliability, and decreased electrical resistivity.

Previously, it has been demonstrated in part that Al or Cu–Ag alloy interconnects can enhance mechanical strength and increase the resistance against electromigration [5,6]. However, alloying normally causes higher electrical resistivity. Fortunately, it is also found that alloying Cu with Ag would cause the increase in the Cu–Ag resistivity the least, when compared to other copper alloys [7,8].

According to Nordheim’s Rule [ρ = ρ_matrix_ + CX(1-X), where X is the element fraction and C is the constant], a thoroughly mixed alloy (single phase) will have a higher resistivity than either one of the pure metals. On the other hand, the phase diagram of Cu–Ag predicts that there might be Ag-rich and Cu-rich phases co-existing in the structure of these alloys. The positive heat-of-mixing between Ag and Cu further explains the formation of a dual-phase structure [9]. This implies that a sputtered Ag–Cu film may separate into two phases upon annealing, although the alloy becoming trapped in either phase is also quite possible. If a dual-phase structure is formed, the alloy’s resistivity would likely follow the mixture rule [ρ = X_α_·ρ_α_ + X_β_·ρ_α_, where X is the volume fraction, ρ_α_ and ρ_β_ are the resistivity of α phase and β phase], which means the resistivity would fall between that of Ag and Cu. Therefore, to fully utilize these alloys, a suitable annealing process must be applied in order to induce the dual-phase structure. The study on mechanical properties of as-deposited Ag–Cu films shows that hardness is a function of Ag:Cu ratios [10]. The hardness values of these alloys ranged from 2 GPa to 4 GPa, which exceeded the hardness values of pure polycrystalline copper (1 GPa–1.5 GPa) and silver (0.7 GPa). However, in that particular study, the effect of annealing was not included. If these alloy films were annealed, the solubility of Ag in Cu or Cu in Ag would be decreased to less than 1% at room temperature [9], and the hardness would drop significantly due to recrystallization. Regarding the grain size, it was reported previously that the alloy films’ grain sizes was much smaller than that of pure Ag or Cu [9], and it was more difficult to grow [11]. More importantly, Sheng et al. [12] found that the annealing temperature can be as low as 230 °C.

Ag_40_Cu_60_ is a eutectic alloy. This implies that, by adjusting the Cu:Ag ratio in a co-sputtered Cu–Ag film, one may be able to observe a much lower annealing temperature than expected. These phenomena could be critical to a device and process engineer.

The Cu–Ag alloy films can be prepared using chemical vapor deposition [1], electrode position [7], and physical vapor deposition [9]. The present study deposited Cu–Ag films with various Cu:Ag ratios on glass substrates using a co-sputtering process. The films’ roughness, structures, mechanical properties, and electrical properties were then characterized as functions of their respective compositions and annealing conditions. Additionally, the elemental dissolution rates for both metals in these films were measured in a buffer solution. This could be essential to the reliability of the devices containing these alloys.

## 2. Results and Discussion

### 2.1. Structural Analysis

**Figure 1:**
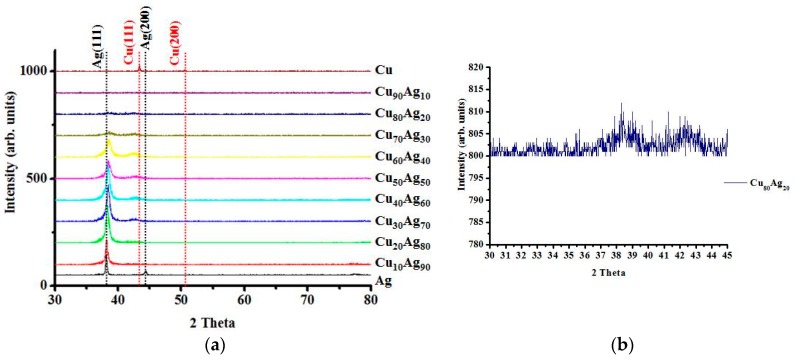
X-ray diffractometry (XRD) patterns of (**a**) as-deposited Cu–Ag films as a function Cu:Ag atomic ratios; (**b**) the enlarged spectrum of sample Cu_80_Ag_20_. It contains Ag (111) and Cu (111) orientations.

**Figure 2:**
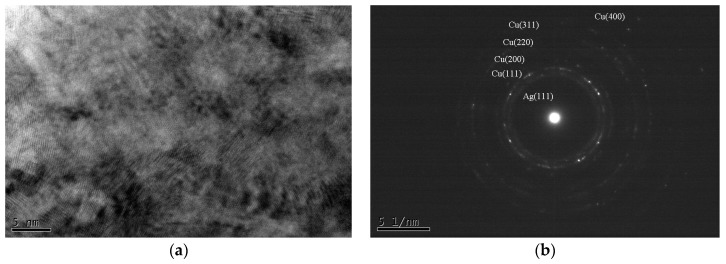
(**a**) Cross-sectional TEM micrograph of a Cu_90_Ag_10_ film; (**b**) its diffraction pattern. The circled areas in (a) represent Cu (111).

The X-ray diffraction patterns of the various Cu–Ag films in the as-deposited state are presented in Figure 1. It can be observed that, regardless of Cu:Ag atomic ratios, the as-deposited films only have one major diffraction peak, either Ag or Cu. In some cases, only one peak/phase can be identified, such as the case of Cu_90_Ag_10_. In Figure 1, the peaks for samples Cu_80_Ag_20_ and Cu_90_Ag_10_ are hard to identify due to data compression. The inset in Figure 1 shows the diffraction pattern of the sample Cu_80_Ag_20_. For sample Cu_90_Ag_10_, only a small Cu (111) peak can be identified. When the Cu_90_Ag_10_ film was further examined with high resolution transmission electron microscopy (HRTEM), nano-crystalline Cu (<10 nm) was easily observed, as shown in Figure 2a. In Figure 2b, a trace of Ag (111) ring was observed, although it is hard to find the image of the Ag phase in Figure 2a. This is not surprising because it is known that Cu can dissolve Ag atoms only up to 4.9 at %, while Ag can dissolve up to 14.1 at % Cu. Therefore, it is quite possible that all the as-deposited alloy films, except for the sample Cu_10_Ag_90_, have a dual-phase structure, and each phase may be supersaturated with another alloy element. The supersaturated condition is caused by the co-sputtering process, which is known to be a non-equilibrium deposition method. This can be observed in Figure 1, where the Ag (111) shifts to the right with the increase in Cu content. It has been reported that a continuous fcc Ag–Cu solid solution, or even an amorphous structure, can be forced to form at low temperatures via vapor deposition [12,13]. It has already been mentioned that the sputter-deposited solid solution of the binary Ag–Cu alloy is unstable, and an initially metastable or nanocrystalline Ag–Cu alloy film will separate into Ag-rich and Cu-rich phases during annealing. This is due to the large miscibility gap and positive enthalpy of mixing for Ag–Cu systems [10,14].

**Figure 3:**
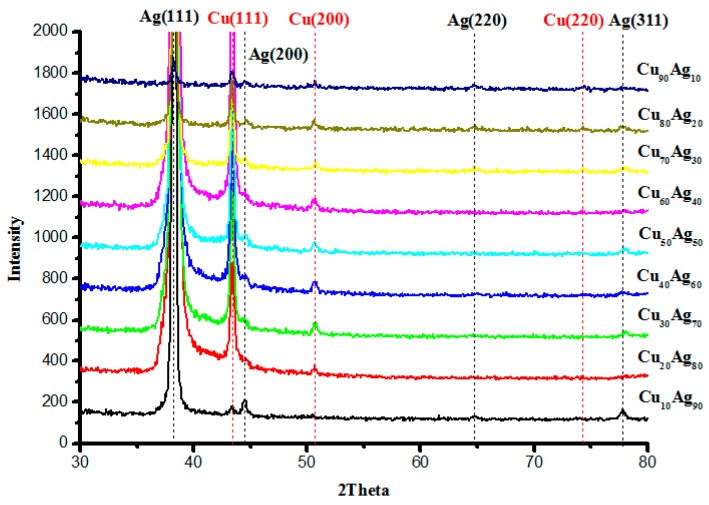
XRD patterns of annealed Cu–Ag films as a function Cu:Ag atomic ratios. (Annealing conditions: 400 °C for four minutes).

**Figure 4:**
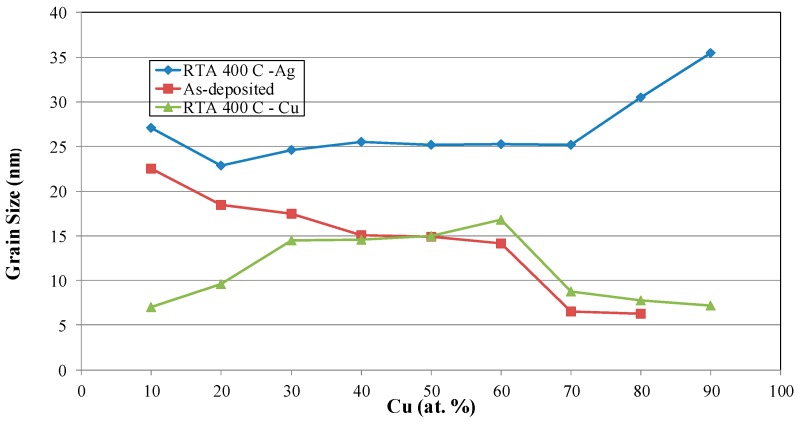
Calculated Ag and Cu grain sizes in Cu–Ag films, before and after annealing.

**Figure 5:**
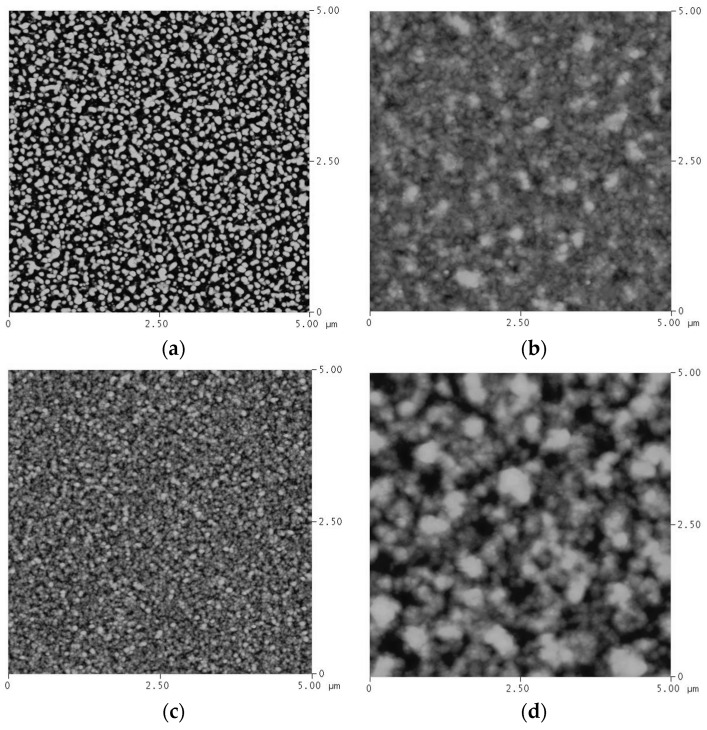
Atomic force microscopy (AFM) surface morphologies of Cu_40_Ag_60_ and Cu_90_Ag_10_ films, before and after annealing: (**a**) as-deposited Cu_40_Ag_60_; (**b**) Cu_40_Ag_60_ after annealing; (**c**) as-deposited Cu_90_Ag_10_; (**d**) Cu_90_Ag_10_ after annealing.

Figure 3 shows the X-ray diffraction patterns for the films annealed at 400 °C for 4 min. It can be seen that all the alloy films show a clear dual-phase structure, with a preferred orientation at (111). The Ag grain size of each film is calculated by applying the Sherrer formula on the peak located near Ag (111). The result is presented in Figure 4. It can be seen from this figure that the Ag grain size of the as-deposited films decreases with the increase in Cu content. The alloying effect on the grain size is normally found on co-deposited metal films due to the positive heat of mixing. It is known that alloying a small amount of element A into element B through sputtering, with low solubility (or immiscibility) between A and B, tends to minimize grain sizes [15]. It has been proven via an in-situ TEM study that the minority element segregates to the surface and stops the growth of the crystal grain [9]. For the films annealed (at 400 °C), it was found that the Ag grain size increases with the increase in Cu content. This implies that, during annealing, the Ag atoms in the over-saturated Cu-rich phase quickly left the Cu-rich phase, resulting in the increased growth rate of Ag grains. According to the Cu–Ag phase diagram, the Cu-rich phase has less solubility for Ag (4.9 at % max.) and a higher melting point (1050 °C) than that of the Ag-rich phase for Cu (14.1 at % and 880 °C). Therefore, during annealing, the trapped Ag atoms should quickly agglomerate into Ag grains and enhance grain growth. On the other hand, the grain growth of Cu is not as obvious. In Figure 4, the calculated Cu grain size after annealing is presented, As observed, the grain growth of Cu occurred when the Ag content is in the range of 30 at %–70 at %. When the samples have a high percentage of Ag, Cu atoms can be dissolved in a Ag matrix. Hence, grain growth will not be as significant as that of Ag. The atomic force microscopy (AFM) study of the surface morphologies of Cu_40_Ag_60_ and Cu_90_Ag_10_ films show consistent results, which are illustrated in Figure 5. It is clearly seen that the grain size of Cu_90_Ag_10_ is larger than that of Cu_40_Ag_60_. The existence of the Cu phase can enhance the growth of the Ag grain. The results of the surface roughness measurements are presented in Table 1. The trend is generally consistent with those shown in Figure 4, if the increase in roughness is related to the grain growth of Ag.

### 2.2. Electrical Properties

**Figure 6:**
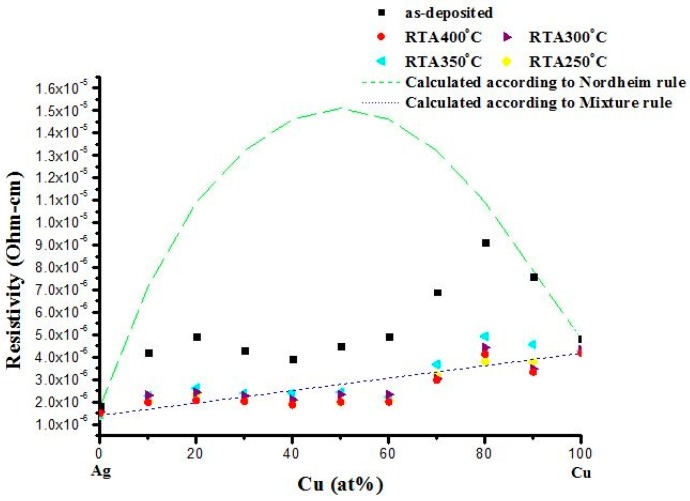
Electrical resistivity of Cu–Ag alloy films, before and after annealing. The calculated resistivity according to Nordheim’s rule and mixture rule are plotted as references.

The resistivity of the films before and after annealing was measured using a four-point probe. The results are presented in Figure 6. Without annealing, the resistivity values show a twin-peak pattern when plotted against composition. The peak locations are near the Cu-rich and Ag-rich ends, which are close to the values calculated using Nordheim’s rule. This means that, in those peaks, the films can be considered to be a thoroughly mixed sample (one phase) after co-deposition. Obviously, when the percentage of the alloy element is less than 10 at %, the Cu-rich or Ag-rich alloy film is much closer to having a single-phase structure, and the minor (alloy) element is mixed into the matrix, most likely in an over-saturated state. The green dash line in Figure 6 is plotted according to Nordheim’s rule. The constant, C, normally depends on the alloying element and the matrix. Here, in Figure 6, the value of C is estimated to be 60 μΩ-cm. When these alloy films were annealed, the Cu-rich and Ag-rich phases were easily separated. In this case, the resistivity values of these films were calculated using the general mixture rule, which is plotted as black dotted line. It is noticed that the resistivity of some alloy films, particularly Cu_40_Ag_60_, Cu_50_Ag_50_ and Cu_60_Ag_40_, is below the simple mixture-rule line. This deviation could be attributed to the grain growth of Cu (as illustrated in Figure 4) in this composition range. These results could be important because they imply that some of the annealed Cu–Ag films can be good candidates in the applications of high-density electronic devices. In high-density electronic devices, electromigration and low electrical resistivity are major concerns. It can also be seen in Figure 6 that the structure of all alloy films can separate into two phases when annealed at temperatures as low as 250 °C using RTA. Being annealed at higher temperatures, the samples may not improve their resistivity further, and the grain size only increases. This should be avoided to maintain good electromigration resistance.

### 2.3. Mechanical Properties

**Figure 7:**
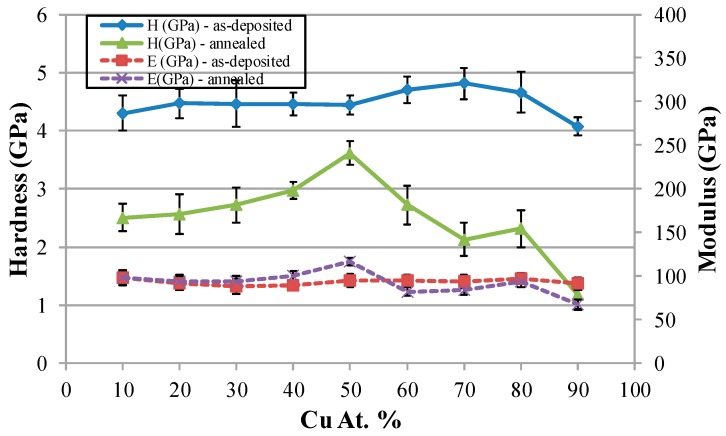
Hardness values and moduli of Ag–Cu alloy thin films before and after annealing.

Figure 7 shows the measured hardness and modulus values of these Cu–Ag alloy films. In the as-deposited conditions, all the films have higher hardness values. After being annealed at 400 °C using RTA, the hardness of these films decreased, as expected. The film with a 50:50 Cu:Ag ratio has the highest hardness after annealing, which could be due to the solution hardening in both phases. For the Cu-rich films (>50%), the hardness decreases with the increase in Cu content. This part is consistent with the annealing effects (recrystallization and grain growth) shown in Figure 4. In sum, the Cu-rich films can be recrystallized faster than Ag-rich films. According to the results of the modulus measurements, it was found that the as-deposited films have a similar modulus, which means that these films have similar elastic behavior or binding strength. After being annealed, the Cu_50_Ag_50_ film has the highest modulus, whereas the Cu-rich film has the lowest modulus value, even though Cu has a higher melting point than that of Ag.

### 2.4. Dissolution Behaviors

**Figure 8:**
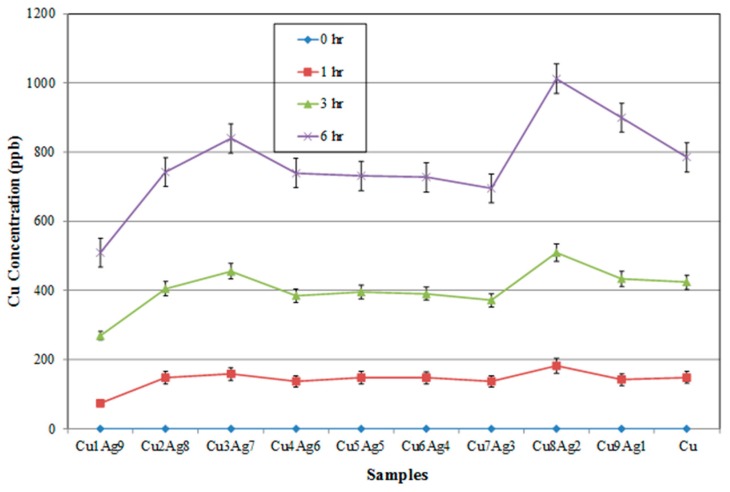
Cu ^+^ concentrations of Cu–Ag alloy films immersed in a buffer solution (pH = 7) as a function of time.

**Figure 9:**
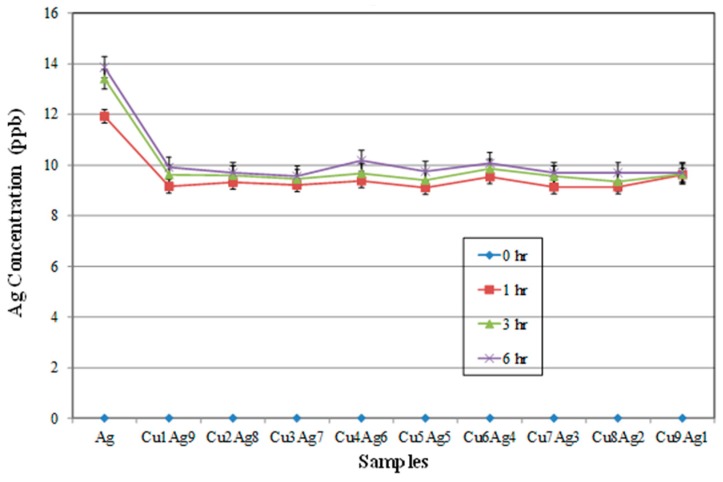
Ag^+^ concentrations of Cu–Ag alloy films immersed in a buffer solution (pH = 7) as a function of time.

It is important to know the corrosion behavior of these alloy films, as this property is related to the reliability of electronic interconnections. From Figure 8 and Figure 9, it can be observed that Cu can be dissolved more easily in a buffer solution than can Ag. According to Figure 8, Cu in alloy films may dissolve faster than pure Cu. This could be due to the galvanic effect between Cu and Ag. Cu is less noble than Ag and thus will dissolve preferentially in most solutions [16]. However, a twin-peak pattern was observed once again. This may have nothing to do with galvanic effects and may be more related to the highly supersaturated states existing in both Cu-rich and Ag-rich phases, which created highly stained structures. This is not uncommon for co-sputtered thin films. Moreover, it can be seen in Figure 9 that all the alloy films have lower Ag-ion concentrations than those of pure Ag after 6 h immersion. This result can be related to the sacrifice of the Cu phase.

## 3. Materials and Methods

The co-sputtering process was conducted with pure copper (99.995%) and silver (99.995%) targets that were both 2 in. in diameter. A glass substrate (Corning 1737) was placed 45° to the target surface with a substrate-to-target distance of 10 cm. The glass substrates were ultra-sonically cleaned with 5% KOH and rinsed in DI water to remove grease and organic contaminants. During deposition, the substrate was kept unheated, and the flow rate of argon was 35 sccm. Before deposition, the chamber was first pumped down to 2.66 × 10^−4^ Pa, and Ar gas was then introduced to fill the chamber up to 0.43 Pa. Prior to film deposition, the target was sputter-cleaned in Ar plasma at a pressure of 0.65 Pa for 20 min, with the target shutter closed. At the same time, the substrates were cleaned in Ar plasma for 10 min by applying a bias of 80 W (RF). In order to prepare a film with proper composition (e.g., Cu_10_Ag_90_), several deposition processes had to be repeated with the target power adjusted right after the examination of the composition using EDS (Energy Dispersive Spectrometry, Xflash, Bruker, Berlin, Germany). The deviation of atomic percentage was controlled within 0.5%. Some of the deposited films were then annealed for four minutes at 400 °C using a rapid thermal annealing (RTA) system (ARTS-150, Premtek, Taipei, Taiwan) in an argon atmosphere, with the ramping rate set at 100 °C/s. The flow rate of Ar was fixed at 2000 sccm during RTA. The thickness of the film was measured using a surface profiler (ET3000, KOSAKA, Tokyo, Japan). The final thickness of the deposited films was 500 nm.

The phases of the deposited films were studied using X-ray diffractometry (XRD). The X-ray diffractometer (PW 1830, Philips, Almelo, The Netherlands) was used at monochromatic high intensity Cu Kα radiation (λ = 0.1541 nm). The scanning angle was from 30° (2θ) to 80° (2θ), with a step size of 0.04° and a measuring time of 1.60 s per step. Atomic force microscopy (AFM, Dimension 3100, Digit Instruments, Santa Barbara, CA, USA) was used to examine the surface morphologies and roughness of the films. The resistivity of the films was measured using a four-point probe. Transmission electron microscopy (JEM2100, JEOL, Tokyo, Japan) was used to examine the detailed microstructure of films.

The mechanical properties (i.e., the hardness and Young’s modulus) of the films were characterized by a Hysitron triboindenter (TI-900, Hysitron, Minneapolis, MN, USA) equipped with a Berkovich tip. Prior to the tests, the tip area function was carefully calibrated following a standard procedure as well documented by Oliver and Pharr [17]. According to Chen and Bull [18], the suggested indentation penetration for most coated systems should be less than one-tenth of the coating thickness to minimize the substrate effect. In this study, the indentation depth was set at 50 nm with a displacement control mode, while the loading rate was set at 1 nm/s. Each sample was indented eight times, and an average value of hardness and reduced modulus was obtained. The hardness and reduced modulus were determined on the basis of the Oliver and Pharr method [17]. The dissolved concentrations of Cu- and Ag- ions of these alloy films when immersed in a buffer solution (pH = 7), as a function of time, were measured using an ICP-Mass (OPTIMA 2100 DV, PerkinElmer Inc., Billerica, MA, USA).

## 4. Conclusions

Cu–Ag alloy films with various Cu:Ag atomic ratios were prepared using a dual-target co-sputtering technique. The structural, mechanical, and electrical properties of these films, before and after annealing, were examined as functions of composition and annealing temperature. The results show that the co-deposited films can have either a one-phase or two-phase structure. A second phase may exist when a critical alloy concentration is reached. After being annealed at 400 °C using RTA, all alloy films have a dual-phase structure with a preferred orientation of [111]. The grain size of the Ag phase in these alloy films changes according to the Cu:Ag atomic ratios. It decreases with the increase in Cu content for the as-deposited samples. After annealing, the grain size increases with the increase in Cu content. This can be explained by the fact that Cu can only dissolve Ag up to 4.9 at %, while Ag can dissolve up to 14.1 at % Cu. The resistivity values of the as-deposited films would follow Nordheim’s rule to a certain extent for over-saturated Cu-rich or Ag-rich samples. When the composition falls in the miscibility gap (two-phase region), the resistivity will decrease. Contributed by both mechanisms, twin-peak characteristics would appear on the resistivity spectrum (distribution) over the whole range of composition. After being annealed, the samples show that the resistivity roughly follows the mixture rule, mainly due to the separation of thermally stable Ag and Cu phases.

Under un-annealed conditions, all films have higher hardness values. Annealing will cause a decrease in hardness. The annealed films, composed of 40 at %–60 at % Cu, have higher hardness and lower roughness than those of other compositions. Obviously, these films have a higher resistance to recrystallization. According to the dissolution test, it was found that the Cu-ion concentration in the buffer solution was about 40 times higher than that of Ag ions. The galvanic effect is observed to accelerate the dissolution of Cu and slow down that of Ag. The over-saturated state may accelerate the dissolution of Cu, which results in twin-peak characteristics.

## Figures and Tables

**Table 1:** Surface roughness of Cu–Ag alloy films before and after annealing.

Samples	As-Deposited (Ra)	RTA 400 °C (Ra)
Cu_10_Ag_90_	3.80 ± 0.29 nm	12.38 ± 1.72 nm
Cu_20_Ag_80_	2.86 ± 0.27 nm	9.37 ± 1.91 nm
Cu_30_Ag_70_	1.82 ± 0.19 nm	9.64 ±1.51 nm
Cu_40_Ag_60_	2.13 ± 0.30 nm	6.06 ± 1.73 nm
Cu_50_Ag_50_	1.98 ± 0.22 nm	7.38 ± 1.06 nm
Cu_60_Ag_40_	1.58 ± 0.25 nm	4.73 ± 1.44 nm
Cu_70_Ag_30_	2.17 ± 0.37 nm	10.29 ± 1.89 nm
Cu_80_Ag_20_	2.33 ± 0.39 nm	11.70 ± 2.23 nm
Cu_90_Ag_10_	2.63 ± 0.32 nm	21.68 ± 2.55 nm
